# Experimental Study to Investigate the Performance-Related Properties of Modified Asphalt Concrete Using Nanomaterials Al_2_O_3_, SiO_2_, and TiO_2_

**DOI:** 10.3390/ma17174279

**Published:** 2024-08-29

**Authors:** Amjad H. Albayati, Nazar K. Oukaili, Mustafa M. Moudhafar, Abbas A. Allawi, Abdulmuttalib I. Said, Teghreed H. Ibrahim

**Affiliations:** Department of Civil Engineering, University of Baghdad, Baghdad 17001, Iraq; nazar.oukaili@coeng.uobaghdad.edu.iq (N.K.O.); mustafa.moudhafar@coeng.uobaghdad.edu.iq (M.M.M.); a.allawi@uobaghdad.edu.iq (A.A.A.); dr.abdulmuttalib.i.said@coeng.uobaghdad.edu.iq (A.I.S.); tagreed.hassan@coeng.uobaghdad.edu.iq (T.H.I.)

**Keywords:** nano alumina, nano silica, nano titanium, moisture damage, permanent deformation, fatigue

## Abstract

The dual nature of asphalt binder necessitates improvements to mitigate rutting and fatigue since it performs as an elastic material under the regime of rapid loading or cold temperatures and as a viscous fluid at elevated temperatures. The present investigation assesses the effectiveness of Nano Alumina (NA), Nano Silica (NS), and Nano Titanium Dioxide (NT) at weight percentages of 0, 2, 4, 6, and 8% in asphalt cement to enhance both asphalt binder and mixture performance. Binder evaluations include tests for consistency, thermal susceptibility, aging, and workability, while mixture assessments focus on Marshall properties, moisture susceptibility, resilient modulus, permanent deformation, and fatigue characteristics. NS notably improves binder viscosity by about 138% and reduces penetration by approximately 40.8% at 8% nanomaterial (NM) content, significantly boosting hardness and consistency. NS also enhances Marshall stability and decreases air voids, increasing the mix’s durability. For moisture resistance, NS at 8% NM content elevates the Tensile Strength Ratio (TSR) to 91.0%, substantially surpassing the 80% standard. Similarly, NA and NT also show improved TSR values at 8% NM content, with 88.0% and 84.1%, respectively. Additionally, NS, NA, and NT reduce permanent deformation by 82%, 69%, and 64% at 10,000 cycles at 8% NM content, illustrating their effectiveness in mitigating pavement distress. Notably, while higher NM content generally results in better performance across most tests, the optimal NM content for fatigue resistance is 4% for NS and 6% for both NA and NT, reflecting their peak performance against various types of pavement distresses. These results highlight the significant advantages of nanoparticles in improving asphalt’s mechanical properties, workability, stability, and durability. The study recommends further field validation to confirm these laboratory findings and ensure that enhancements translate into tangible improvements in real-world pavement performance and longevity.

## 1. Introduction

Asphalt binder, a critical component in asphalt concrete mixes for paving applications, faces significant challenges due to its dual nature: it exhibits elastic solid behavior under low temperatures or rapid regime of loading; under elevated temperatures or slow regime of loading, it exhibits a viscous fluid behavior. This duality creates a need to improve the performance of asphalt binder to resist fatigue stresses, leading to cracking in cold weather conditions and plastic deformation (rutting) in hot climatic conditions. Traditionally, improvements have involved the use of additives like polymers, waste materials, fibers, and fillers; however, these modifications have not fully addressed the performance shortages. Recently, the advent of nanomaterials, which have at least one dimension within the 1 to 100 nanometer range, has offered promising solutions due to their unique physicochemical properties and high surface area [1].

Nano alumina (NA) is relatively widely available due to the abundant natural occurrence of its raw material, bauxite, from which aluminum oxide is derived. NA is the perfect material for demanding applications in engineering and high-performance materials because of its exceptional heat conductivity, large surface area, excellent mechanical strength, high stiffness, and ability to withstand oxidation [2]. Mubaraki et al. [3] examined the impact of incorporating ASA polymer and NA nanoparticles into asphalt concrete at concentrations of 3%, 5%, and 7%. Their results showed that asphalt cement’s rheological properties may be significantly improved at elevated temperatures. Of them, the five percent Nano Alumina combination showed the greatest increase in resistance to rutting. Following this, Al-Mansob et al. [4] explored the use of NA as an additive in epoxidized natural rubber (ENR) modified asphalt for improving rutting resistance. Their Dynamic Shear Rheometer test results affirmed that NA boosts the high-temperature resistance of bitumen. The effects of NA on SMA asphalt mixtures’ fatigue resistance were evaluated by Chelovian and Shafabakhsh [5], identifying 0.6% as the optimal concentration for maximizing the lifespan of the mix. More recently, Bhat and Mir [6] conducted studies on the usage of NA in SBS-modified bitumen, testing concentrations from 1% to 5%. Their research indicated improvements in fatigue performance up to the 4% level, although increasing the NA content to 5% resulted in increased fatigue issues. The remarkable physical–mechanical properties of NA, such as its high surface area, considerable heat conductivity, and excellent strengths, were repeatedly emphasized in these studies as essential aspects of improving the stability and performance of asphalt binders in a range of scenarios.

NS, or Nano Silica, has a wide range of uses in several fields, making it one of the most widely utilized nanomaterials, including engineering [7,8], and its low preparation cost as well as high performance [9]. NS disperses effectively in mixtures and has significant adsorption, substantial surface area, and high pozzolanic behavior. However, because of its substantial capability for adsorption and significant surface area, several studies have highlighted possible problems with agglomeration during mixing [10,11]. NS has been found to improve rheological properties and oxidative aging resistance, impacting both fatigue and rutting resistance [12,13]. NS increases the rotational viscosity and softening point of asphalt, though it may influence low-temperature performance [14]. Yusoff et al. [15] demonstrated that 4% NS significantly improves the aging resistance of polymer-modified asphalt. Taherkhani and Tajdini [16] indicated that NS is more effective than hydrated lime in enhancing asphalt concrete properties. Badroodi et al. [17] observed that NS nanoparticles enhance the rheological behavior and aging resistance of bitumen, with 1.2% NS providing optimal performance. Long et al. [18] found that NS reduces aging sensitivity and moisture damage, slightly increasing bitumen adhesion. Kamboozia et al. [19] found that 6% NS and 50% RAP provide the best rutting resistance in SMA mixtures. Furthermore, the authors noted that 4% NS offers the best fatigue resistance, while higher concentrations reduce fatigue life. The application of 4% NS, according to Albayati et al. [20], might enhance the aging resistance of asphalt binder, increasing its rutting factor and stiffness and making it appropriate for high temperatures and high traffic circumstances.

Nano Titanium Dioxide (NT) is a photocatalytic compound that absorbs organic and inorganic air pollutants, making it suitable for coating pavements [21]. The physical characteristics of bitumen, such as ductility, penetration, and softening point, are enhanced by the introduction of Nano Titanium Dioxide [22,23]. NT has also shown varied improvements depending on the dosage used. Shafabakhsh et al. [22] found that NT nanoparticles enhance the rheological behavior and aging resistance of bitumen, with 0.9% NT providing optimal performance. Hu and Yu [24] found that NT and thermochromic powder increase moisture resistance in HMA mixtures. The chemical, rheological, and morphological improvements of asphalt binders following the incorporation of nanomaterials have been clarified by thorough, intensive investigations employing rheological assessment, SEM, and FTIR. Using experimental data and numerical methods of assessments under different conditions, the creep and fatigue performance of both conventional and nano-modified asphalt mixtures have been anticipated, demonstrating significant improvements brought about by NT [25,26,27]. Albayati et al. [20] concluded that NT uniquely enhances high-temperature performance and storage stability. Furthermore, they came to the conclusion that 4% of NT is the optimal dosage for fatigue resistance since it provides a balanced modification that enhances stiffness without appreciably shortening the binder’s fatigue life.

Overall, the increasing use of NA, NS, and NT has substantially influenced the physical as well as rheological behavior and aging resistance of the asphalt binder, thus enhancing pavement resistance to rutting and fatigue [20,27,28,29,30]. Extending beyond previous research, this study investigates the effects of NA, NS, and NT not only on asphalt binder properties but also on asphalt concrete mixtures. It examines the comprehensive impacts of these nanomaterials at the mixture level, focusing on Marshall properties, moisture susceptibility, resilient modulus, permanent deformation, and fatigue characteristics, providing a comprehensive analysis of the potential improvements offered by these nanomaterials and setting the stage for more resilient and durable asphalt pavements.

## 2. Materials

In the experimental phase of this study, materials were selected with an emphasis on local availability and relevance to ensure the integrity of our findings. This included asphalt cement, aggregate, filler, and three types of nanomaterials: NA, NS, and NT. The asphalt cement graded 40/50 penetration value employed in this investigation was produced by the Dora refinery, located southwest of Baghdad, Iraq. Table 1 displays the physical properties corresponding to this asphalt cement, which meets the requirements of the AC 40/50 specification according to guidelines from the State Corporation of Roads and Bridges [31]. Crushed quartz aggregates were selected for the purpose of this work. They separated into coarse and fine fractions using a sieve of 4.75 mm size (No. 4) before being recombined to meet the D-5 mix type specifications as per the ASTM D3515 [32].

The nominal maximum size of aggregate, intended for the wearing course mix type, is 12.5 mm (½ inch), in compliance with ASTM D3515 [32]. The chosen aggregate gradation is meticulously outlined in Table 2. Furthermore, Table 3 offers a comparative analysis of the aggregates’ physical characteristics against the specified standards, confirming their compliance with the prescribed acceptable limits.

Limestone dust mineral filler was used in the preparation of the asphalt mixture. Table 4 displays their physical characteristics and chemical composition.

**Figure 1:**
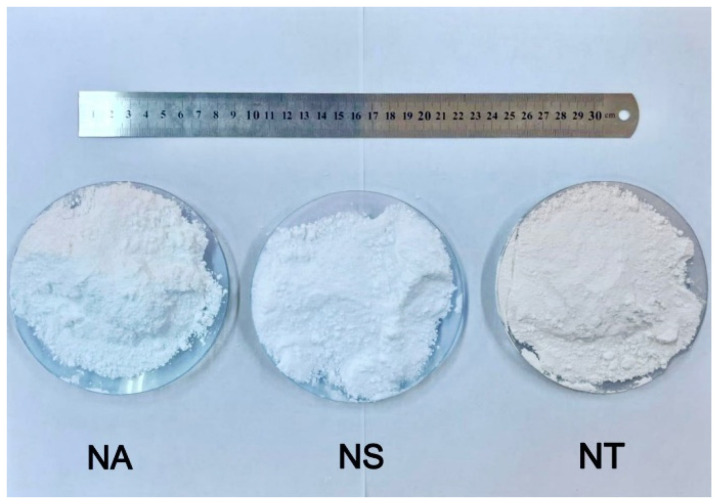
Photograph for nanomaterials.

Three types of nanomaterial were implemented: NA, NS, and NT. Table 5 provides a summary of these nanomaterials’ physical properties, indicating that each possesses particle sizes under 100 nm, thereby categorizing them as nano-scale materials. To offer a visual perspective, Figure 1 showcases photographs of these nanomaterials, illustrating their physical appearance.

**Figure 2:**
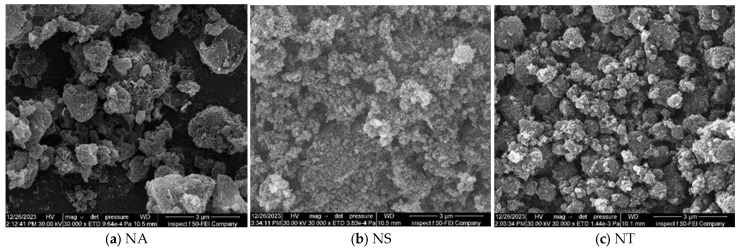
SEM for nanomaterials.

The three nanomaterials employed in this investigation, NA, NS, and NT, are shown in Figure 2 as Scanning Electron Microscope (SEM) pictures, all magnified at 30,000 times their original size. NA exhibits a rough and irregular surface texture with significant agglomeration, indicating its potential for enhancing mechanical interlocking within the asphalt matrix. In contrast, NS particles display a finer and more uniform texture, suggesting their ability to achieve a more homogeneous dispersion throughout the asphalt. NT is characterized by its consistent size and spherical shape, with smooth surfaces that facilitate even distribution within the mixture. These visual indicators from the SEM images provide insights into how each nanomaterial might integrate into and affect the structural properties of asphalt mixtures.

## 3. Nanomaterial Addition Method

Throughout the investigation’s testing stage, the introduction of nanomaterials to 40/50 penetration-grade asphalt cement was carefully conducted to ensure even distribution throughout the binder. Utilizing a high-speed shear mixer, the nanomaterials were incorporated into the asphalt cement in dosages of 0, 2, 4, 6, and 8% by weight. The process began by heating the asphalt cement to 140 °C. After that, the nanomaterials were carefully introduced at an uninterrupted rate of four grams a minute for 20 min; all the while, the mixing was continually maintained at 4000 rpm. This meticulous approach was crucial to ensure that the nanoparticles were uniformly dispersed throughout the binder, which is necessary to improve the asphalt cement’s overall performance.

## 4. Test Matrix

### 4.1. Binder Tests

In this research, various standardized tests were carried out on asphalt binders to assess their performance characteristics. To evaluate the consistency of the binder, the Penetration Test [33] was utilized. This test determines the extent to which a standard needle can pierce asphalt binder in certain circumstances. To assess the thermal susceptibility of the binder, indicative of its performance at elevated service temperatures, the Softening Point Test was performed according to [34]. With the use of the Rolling Thin Film Oven Test, the binder’s resistance to volatile loss under heat was determined [46]. Additionally, the Storage Stability Test [47] was used to evaluate the binder’s ability to retain its consistency following a period of storage. Lastly, the Rotational Viscosity Test [48] at 135 °C helped analyze the binder’s flow characteristics and workability, crucial for its application in field conditions

### 4.2. Mix Tests

In accordance with ASTM standards, extensive testing was performed on asphalt concrete mixtures to assess their performance. By employing cylindrical specimens with a diameter of 101.6 mm and a height of 63 mm, the Marshall test [49] was utilized to evaluate the resistance to plastic deformation. It examined volumetric features such as air voids, voids filled with asphalt, stability, flow, and theoretical specific gravity. Moisture susceptibility tests [50,51] involved the same-sized specimens prepared to a specific air void content (6–8%) and subjected to simulated wet and dry environments, followed by tensile strength assessments. Furthermore, uniaxial repeated load tests were used to determine resilient modulus and permanent deformation on cylindrical specimens with a diameter of 101.6 mm and height of 203.2 mm, where compressive loads were applied monotonically to evaluate the material’s response to stress. Fatigue resistance was assessed using the Indirect Tensile Cracking Test (IDEAL-CT, ASTM D8225 [52]), conducted on specimens with a height of 101.6 mm and diameter of 63 mm in diameter, compacted to around 7% air void level using a Marshall compactor, applying consistent vertical loads and analyzing the resultant load-displacement data. For the purpose of using asphalt concrete mixtures in pavement engineering, these tests provided a full understanding of the durability and mechanical characteristics of the mixtures.

### 4.3. Mix Design

**Figure 3:**
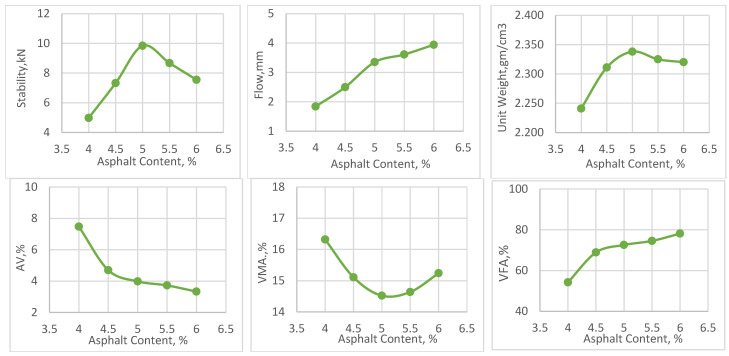
Marshall mix design plots.

The Marshall mix design method was employed to determine the optimum asphalt content (OAC) for mixtures containing neat asphalt. Asphalt contents ranging from 4.0% to 6.0% by total mix weight, in increments of 0.5%, were methodically assessed to establish the OAC. This was achieved by averaging three values: the asphalt content at maximum unit weight, at maximum stability, and the content achieving 4% air voids, as depicted in Figure 3. It was found that 5.0% was the optimal binder percentage for achieving the preferred properties of the mixture, which included flow between 2 and 4 mm, Voids in Mineral Aggregate (VMA) above 14%, and Voids Filled with Asphalt (VFA) between 70 and 85%. To ensure that the variations in the properties of the compacted mixtures were primarily due to the type and content of nanomaterials, the obtained OAC was kept constant for all other mix types.

## 5. Experimental Results and Analysis

### 5.1. Binder Consistency and Thermal Susceptibility

**Figure 4:**
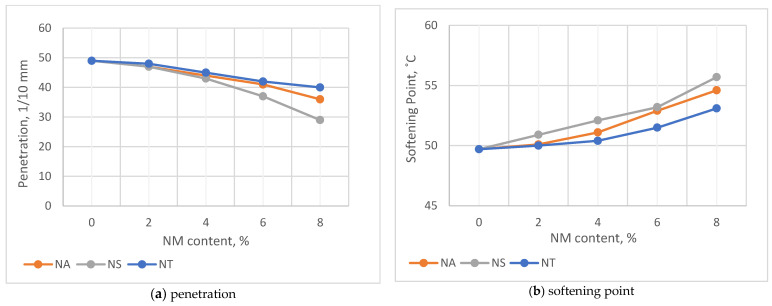
Impact of NM on penetration values (**a**) and softening point (**b**).

Figure 4a,b exhibit the consistency and thermal susceptibility of asphalt binders modified with different nanomaterials: NA, NS, and NT. The Penetration Test results, a measure of consistency shown in Figure 4a, indicate that NS significantly enhances binder hardness by reducing penetration by approximately 40.8% at an 8% addition, compared to the reference asphalt without nanomaterials. NA and NT also improve hardness, though to a lesser extent, with reductions in penetration values of 26.5% and 18.4%, respectively. This significant disparity underscores NS’s superior ability to increase binder hardness because of its large surface area and efficient particle interaction. In contrast, the modest reductions with NA and NT suggest less interaction with the binder matrix, highlighting how the physical and chemical characteristics of each nanomaterial influence their efficacy in improving asphalt binder performance.

Figure 4b focuses on thermal susceptibility, observed through changes in the softening point, which indicates the temperature when the asphalt starts to become less viscous. The results demonstrate a clear trend of increasing softening points with higher NM content, suggesting improved performance in hotter conditions. Specifically, at an 8% NM content, NS exhibits the most significant increase in softening point, rising by 12.1% from a baseline of 49.7 °C to 55.7 °C. NA follows with a 9.9% increase to 54.6 °C, while NT records a 6.8% rise to 53.1 °C. These differences highlight the varying degrees of efficacy with which each nanomaterial interacts with the asphalt matrix, particularly under thermal stress. NS’s pronounced impact, likely due to its extensive surface area and particle–matrix interaction, effectively enhances the asphalt’s high-temperature stiffness.

### 5.2. Binder Aging and Storage Stability

**Figure 5:**
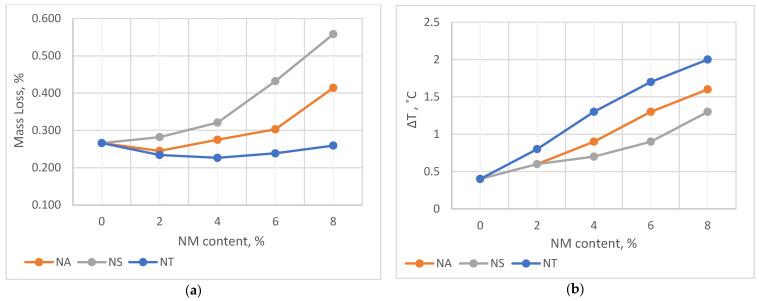
Effect of NMs on (**a**) mass loss and (**b**) storage stability.

The aging resistance of asphalt binders treated with NA, NS, and NT is evaluated by the Rolling Thin Film Oven test (RTFO), the results of which are shown in Figure 5a as mass loss. Remarkably, NT-modified binders exhibit the lowest mass loss, especially notable at an 8% NM content, where it demonstrates a noteworthy 3% reduction in mass loss in comparison to the control binder. The reduction is explained by NT’s high colloidal weight per unit volume and its ability to form a compact network within the binder, effectively minimizing the volatilization of the binder’s aromatic components and thus enhancing its thermal and oxidative stability.

Figure 5b explores the storage stability, which measures how well the binder’s homogeneity is maintained in storage tanks or during transportation. As the NM content increases, there is a noticeable increase in storage stability values, with NT showing the most substantial increase. Although NT’s density might facilitate sedimentation within the binder matrix, its storage stability increase remains below the specification limit of 2.2 °C, suggesting a controlled stabilization that does not compromise the binder’s performance. In contrast, Nano Silica (NS) demonstrates the best performance in terms of storage stability. Despite the general trend of increasing stability values, NS maintains the lowest ΔT value, indicative of minimal thermal susceptibility and better uniformity in the binder mixture. This superior performance of NS is likely due to its lower density and its ability to achieve a more homogeneous dispersion throughout the asphalt, as evidenced by SEM images showing NS’s finer and more uniform texture.

### 5.3. Binder Workability

The Rotational Viscosity Test results, detailed in Table 6, assess the workability as well as flowability of asphalt binders treated with NA, NS, and NT at 115 °C, 135 °C, and 155 °C. Both the binder in its original condition and after short-term aging utilizing the Rolling Thin Film Oven (RTFO) test are given these results. Particularly at lower degrees of temperature, NS considerably stiffens the asphalt binder. This effect is most pronounced in the NS-modified binder, where at an 8% NM content, there is an astounding increase in viscosity of approximately 138% at 115 °C from the original reference binder, which is substantially higher compared to increases seen with NA and NT. After RTFO aging, NS continues to show a remarkable increase of about 135% at the same temperature, underscoring its superior aging resistance. NA and NT also show increased viscosity with added nanomaterial content, but to a lesser extent. At 8% NM content, NA shows an increase of about 59% in viscosity at 115 °C compared to the reference binder and approximately 49% increase after RTFO aging. NT exhibits an increase of approximately 32% at 115 °C and about 48% after RTFO aging, compared to the reference binder.

**Figure 6:**
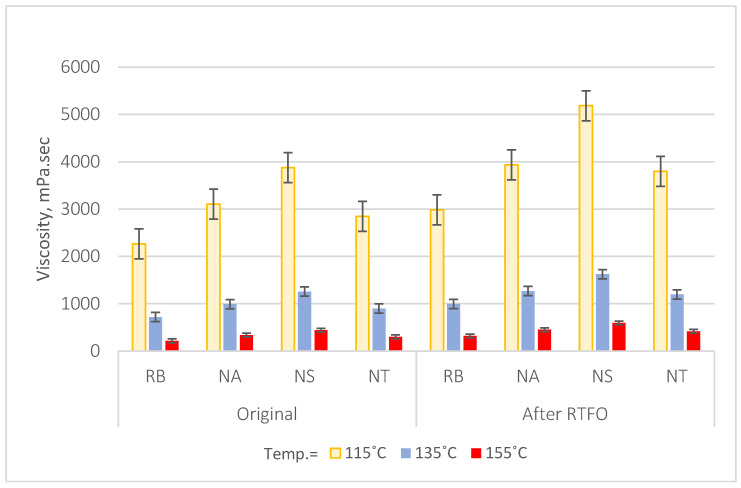
Effect of NM types on asphalt binder viscosity.

To visually represent these effects, data have been sorted by NM type with average viscosity values calculated across all NM content levels, as depicted in Figure 6. This figure illustrates that while NS significantly enhances binder stiffness, especially at lower temperatures, this capability decreases as the temperature rises. NA and NT also improve the asphalt binder’s viscosity and aging resistance, though not as markedly as NS.

These findings highlight the role of nanomaterial type and content in enhancing the workability and durability of asphalt binders, with NS standing out for its robust performance that is particularly advantageous in applications demanding high durability against thermal and mechanical stresses.

### 5.4. Marshall Properties of Mixes

**Figure 7:**
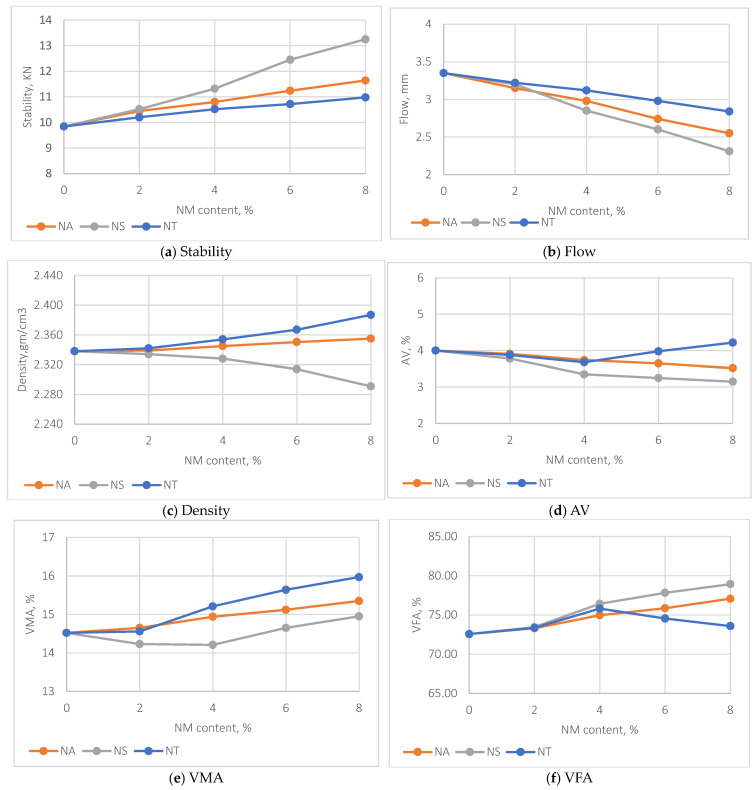
Impact of NM on Marshall properties.

The Marshall properties results, as depicted in Figure 7a–f, reveal that the inclusion of nanomaterials NS, NA, and NT significantly enhances the performance properties of asphalt mixtures, each demonstrating distinct trends. NS notably shows a significant increase in stability from 0% to 8% NM content, with a more pronounced improvement observed between 4% and 8% compared to the 0% to 4% range. This suggests a threshold effect where the benefits of NS in enhancing mix stability become increasingly significant at higher concentrations, exhibiting a 20% improvement at 4% NM content and a 35.6% improvement at 8% NM content compared to the control mix. In contrast, NA and NT exhibit more consistent and linear stability enhancements. NA shows a 19.3% increase, and NT shows an 11.2% increase in stability at 8% NM content, indicating a predictable relationship between nanomaterial content and asphalt stability. The flow values also reflect the stiffening effects of the nanomaterials, particularly NS, which decreases flow by 34.2% at 8% NM content, substantially enhancing the mix’s resistance to deformation while maintaining workability within the desirable 2–4 mm range. NA and NT also decrease flow values by 29.5% and 31.2%, respectively, at the same NM content, confirming increased stiffness.

The density trends, particularly with NS, indicate a slight reduction of 2% at 8% NM content, attributable to its lower density compared to other nanomaterials. Notably, there is a distinct decrease in density at 4% NS content, suggesting an onset of material-specific interactions influencing compaction. In contrast, NA and NT, where the density of mixes increases by 2.7% and 4.2% at 8% NM content, respectively, suggest better particle packing within the mix.

The analysis of air voids (Avs) in asphalt mixtures modified with NA, NS, and NT (Figure 7d) reveals distinct behaviors as nanomaterial content increases. For mixes with NA and NT, air void results are comparable up to NM content of 4%. After this, the trends diverge: AV values for NA slightly decrease, while for NT, they increase. At 8% NM content, NA shows a decrease in Avs by approximately 2.7% compared to the control mix, whereas NT shows an increase in Avs by about 4.3%. Conversely, mixtures incorporating NS demonstrate a steady decrease in air void values at all NM contents, underscoring the binder’s enhanced ability to fill void pockets effectively when modified with NS. By the 8% NM content, NS shows a reduction in Avs by about 5.1% compared to the control mix. This trend is supported by the Voids in Mineral Aggregate (VMA) and Voids Filled with Asphalt (VFA) trends shown in Figure 7e and Figure 7f, respectively.

The VMA values for NS-containing mixes are lower than those for mixes with NA and NT, indicating a stiffer aggregate structure with less space for the binder. Despite this, the VFA trends for NS are higher, suggesting a more effective binder ability in filling the voids. This indicates that even with a stiffer binder, NS can maintain effective coverage and adhesion within the mix, contrasting with NA and NT, where the VFA trends suggest varying degrees of binder efficiency to infiltrate within the pores in aggregate structure. Notably, at 4% NT content, there is a marked decrease in VFA values, indicating a trend of increased air void content. This reduction in VFA suggests that the voids in the mineral aggregate are being filled with air rather than the binder, pointing to the need for further compaction.

### 5.5. Moisture Susceptibility of Mixes

**Figure 8:**
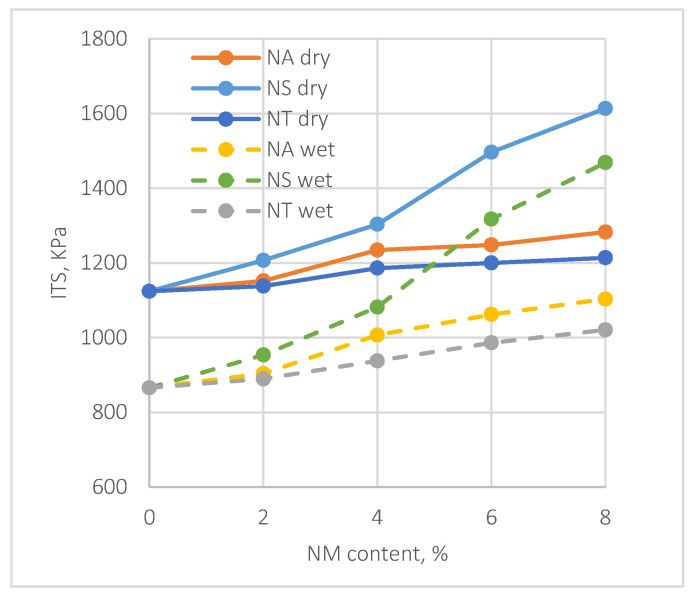
Effect of NMs on ITS.

**Figure 9:**
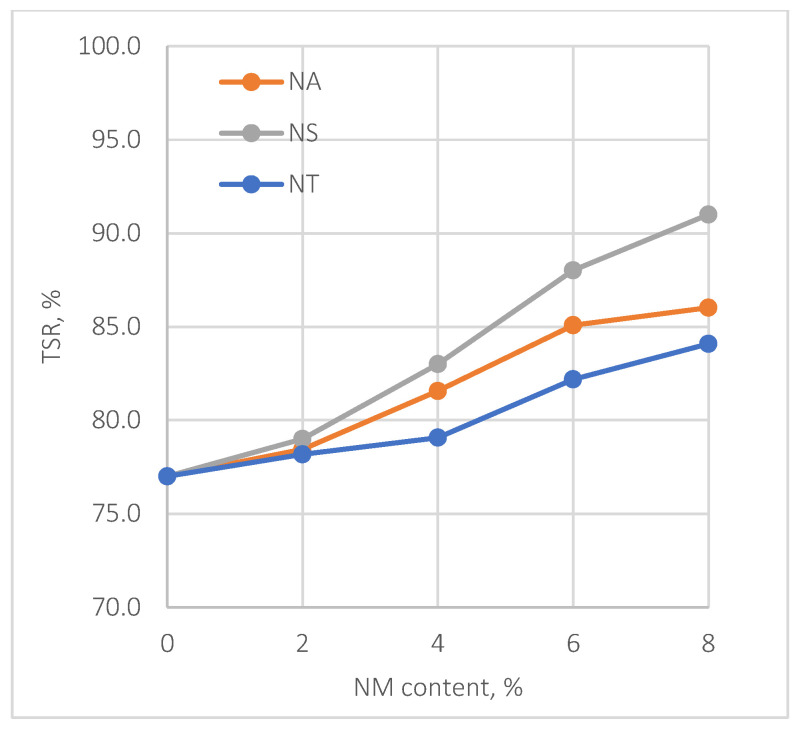
Effect of NMs on TSR.

The evaluation of moisture susceptibility in asphalt mixtures is crucial for understanding their durability. This is typically assessed through the Indirect Tensile Strength (ITS) and Tensile Strength Ratio (TSR) under varying nanomaterial (NM) content levels, as shown in Figure 8 and Figure 9.

Figure 8 indicates that ITS values under dry conditions increase notably with the addition of NMs. Notably, NS shows a significant improvement in dry ITS, peaking at an 8% NM content with a 47.3% increase compared to the control mix, highlighting its substantial enhancement of the asphalt’s tensile strength, especially at higher NM contents. NA and NT also show notable improvements at 8% NM content, with increases of 42.1% and 38.7%, respectively, though slightly less pronounced than NS. Under wet conditions, all nanomaterials improve ITS, albeit less dramatically than under dry conditions. At 8% NM content, NS leads with a 33.5% improvement in wet ITS over the control mix, with NA and NT exhibiting increases of 28.2% and 25.9%, respectively. These increments, while modest compared to dry condition improvements, underscore the nanomaterials’ efficiency in improving the asphalt mixtures’ resistance to moisture.

Figure 9 shows that TSR values assess the asphalt’s ability to resist moisture-induced damage. The control mix fails to meet the 80% specification limit, but the addition of NMs significantly improves this susceptibility. At 8% NM content, NS achieves a TSR of 91.0%, markedly surpassing the required threshold. NA and NT also show improvements, reaching TSRs of 88.0% and 84.1%, respectively, both exceeding the minimum standard, highlighting their effectiveness in enhancing moisture resistance.

The enhanced performance of asphalt mixes containing nanomaterials compared to the control mix is primarily due to the asphalt binder’s enhanced stiffness. This stiffness is crucial during the Indirect Tensile Strength test, enabling the binder to effectively mobilize and withstand tensile forces along the horizontal plane of the specimen, resulting in higher TSR values. Essentially, the addition of nanomaterials increases the binder’s stiffness, significantly enhancing the mix’s ability to resist moisture-induced damage and improving the overall durability of asphalt pavement.

### 5.6. Resilient Modulus of Mixes

**Figure 10:**
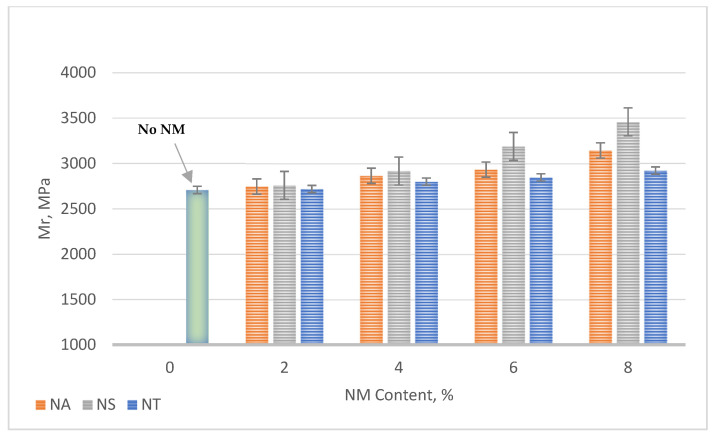
Effect of NMs on resilient modulus.

Figure 10 presents the resilient modulus (Mr) results for asphalt mixes enhanced with nanomaterials (NMs) such as NA, NS, and NT. The resilient modulus is an essential measure of a pavement’s stiffness and its ability to recover after being subjected to traffic loads.

The outcomes demonstrate that the addition of nanomaterials consistently enhances the stiffness of the asphalt mixes, indicated by an increase in Mr values across all types of nanomaterials as their content increases. Specifically, NS leads with a remarkable 28% improvement in Mr at 8% NM content in comparison to the reference mix (i.e., 0% NM content), suggesting a significant enhancement in the pavement’s structural integrity and load-bearing capacity. NA also shows a substantial increase in Mr, with a 16% improvement at 8% NM content, enhancing the asphalt’s stiffness and overall robustness. NT demonstrates a more modest but still notable Increase, Improving Mr by 8% at 8% NM content. These improvements highlight the effectiveness of incorporating nanomaterials into asphalt mixes, not only in strengthening the mix’s internal cohesion and aggregate–binder bonding but also in extending the pavement’s operational life by improving its ability to endure repeated traffic loads. This underscores the potential of nanomaterials like NS, NA, and NT in paving applications to produce more durable and resilient road surfaces, essential for coping with the demands of heavy and continuous traffic.

### 5.7. Mixes Permanent Deformation Results

**Figure 11:**
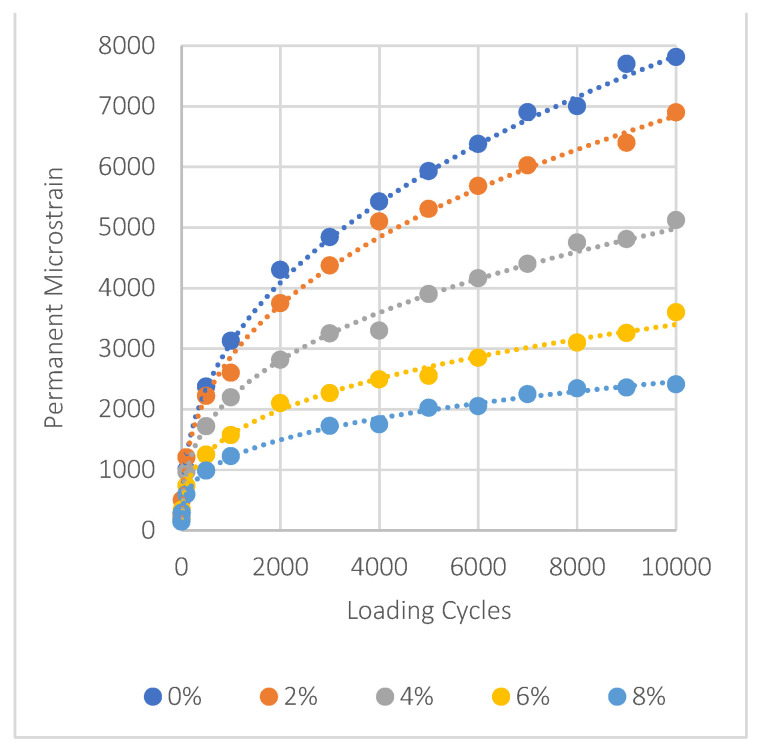
Impact of NA on permanent deformation.

**Figure 12:**
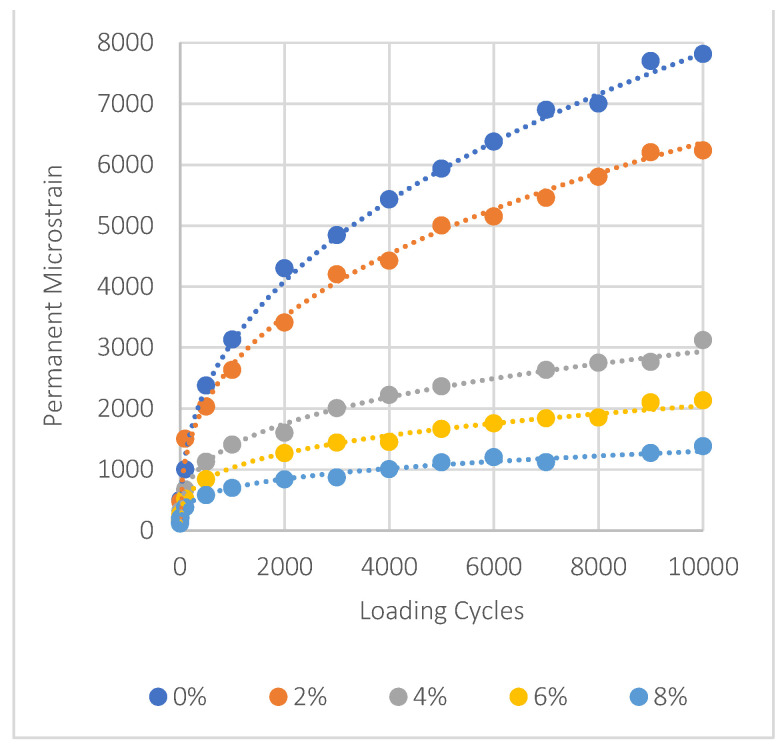
Impact of NS on permanent deformation.

**Figure 13:**
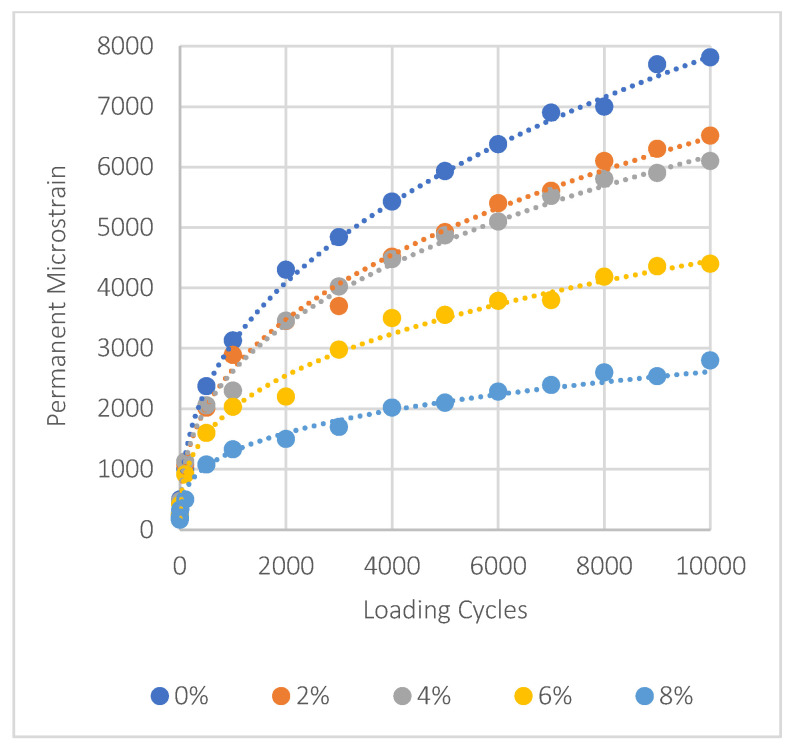
Impact of NT on permanent deformation.

In asphalt mixtures treated with nanomaterials NA, NS, and NT, the evaluation of permanent deformation is shown in Figure 11, Figure 12 and Figure 13. It can be seen that adding more of these materials significantly decreases permanent deformation, which is an important aspect of improving pavement durability. The key factors of permanent deformation are shown quantitatively in Table 7, which is based on data obtained from a power fitting for the logarithmic connection between loading cycles and permanent deformation. These comprise the intercept (‘a’), which represents the initial permanent strain, and the slope (‘b’), which shows the strain accumulation rate. To evaluate the resistance of the mixture to permanent deformation, it is essential to consider these three parameters: ‘a’, ‘b’, and the permanent strain ($\varepsilon_{p}$) at the 10,000th load cycle. Lower values of these parameters indicate stronger resistance to rutting.

For NA, the intercept ‘a’ reduces from 201 at 0% NMs to 142 at 8% NMs, and the slope ‘b’ decreases from 0.397 to 0.312, showing a decline in the strain accumulation rate. This trend is evident in the significant reduction in permanent strain at 10,000 cycles, which drops by approximately 69% compared to the control mix. Similarly, NS shows dramatic improvements, with an 82% reduction in permanent strain at the same load cycle when NM content increases to 8%. NT also exhibits a notable decrease, with a 64% reduction in permanent strain at 8% NMs.

These results underscore the effectiveness of using nanomaterials like NA, NS, and NT to improve the pavement’s resistance to permanent deformation and overall durability. The significant decreases in permanent strain demonstrate how these nanoparticles may significantly increase the service life of asphalt pavements subjected to repeated loading scenarios, offering an affordable solution to improve pavement life span.

### 5.8. Mixes Fatigue Results

**Figure 14:**
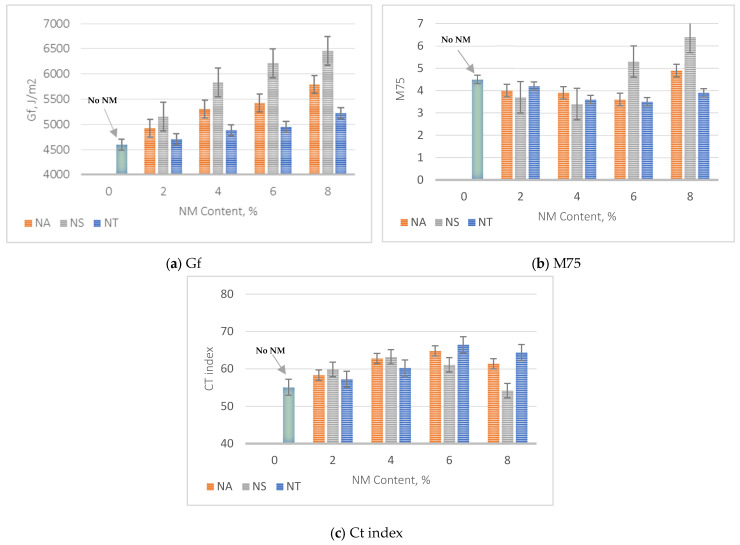
Impact of NMs on fatigue cracking results.

The evaluation of fatigue resistance parameters in asphalt mixtures incorporating nanomaterials NA, NS, and NT, illustrated in Figure 14a–c, highlights distinct trends at varying concentrations. The Gf value, which quantifies the mixture’s resistance to crack initiation, demonstrates that NS mixtures achieve the most significant enhancement. At 8% NM content, NS exhibits a 40.5% increase compared to the control mix, underscoring its superior ability to stiffen the asphalt binder. In contrast, NA and NT show increases of 26.1% and 13.7%, respectively, indicating a relatively lower impact on crack initiation resistance. In terms of M75, which measures resistance to crack propagation (with lower values indicating better performance), NS also shows notable effectiveness, with a 24.4% reduction at 4% NM content. NA and NT also display improvements, with reductions of 20.0% and 22.2% at 6% NM content, respectively. This suggests that while all three nanomaterials contribute to enhanced crack resistance, NS is particularly effective at earlier content levels.

The overall fatigue resistance measured by the CT index is shown in Figure 14c. For NT, the CT index shows a peak at 6% NM content, reaching the highest value among the nanomaterials, indicating the most significant improvement in fatigue resistance at this concentration. The data suggest an approximate 20.5% improvement in fatigue resistance over the control mix, underscoring NT’s effectiveness in enhancing the asphalt mixture’s ability to resist fatigue-related damage under repetitive loading. NA follows closely behind NT, showing its highest fatigue resistance improvement at the same 6% NM content, with an approximately 17.5% increase over the control mix. This enhancement indicates NA’s contribution to improving the asphalt mix’s durability against fatigue cracking. In contrast, NS demonstrates a different trend, achieving its optimal performance at a lower NM content of 4%, where it shows a 14.6% improvement in the CT index compared to the control mix. This result highlights NS’s efficacy at lower concentrations for enhancing fatigue resistance, suggesting that NS might be impacting the asphalt mix’s properties differently compared to NA and NT. This comprehensive analysis demonstrates that specific nanomaterial concentrations can significantly optimize the fatigue resistance of asphalt mixtures. By integrating nanomaterials at these critical concentrations, the durability and longevity of asphalt pavements are greatly enhanced, promoting a more sustainable infrastructure capable of withstanding the fatigue cracking that is mostly caused by strain at the base of the asphalt concrete layer.

## 6. Conclusions

The following significant findings have been drawn from the comprehensive experimental outcomes of asphalt binders and asphalt concrete mixes treated with nanomaterials NA, NS, and NT at different concentrations from 0% to 8%:

1. The SEM analysis of nanomaterials NA, NS, and NT reveals distinctive particle characteristics. NS particles, with their finer and more uniform texture, suggest a potential for more homogeneous dispersion within the asphalt, thereby enhancing the mechanical properties of the mixes;
2. NS significantly enhances the consistency of asphalt binders, reducing penetration by approximately 40.8% and increasing viscosity by about 138% at 8% NM content compared to the reference binder. Conversely, NA and NT also improve binder hardness but to a lesser extent, with penetration reductions of 26.5% and 18.4%, respectively;
3. NT demonstrates substantial improvements in aging resistance, exhibiting the lowest mass loss of 3% at 8% NM content in the RTFO test, indicating enhanced thermal and oxidative stability. NS shows superior performance in storage stability by maintaining the lowest ΔT value, suggesting minimal thermal susceptibility and improved binder uniformity;
4. Nanomaterials significantly improve the Marshall properties of asphalt mixtures. NS notably enhances stability by 35.6% at 8% NM content, demonstrating significant benefits at higher concentrations. NA and NT show consistent stability enhancements of 19.3% and 11.2%, respectively, at the same concentration. Modifications with NS also lead to a consistent reduction in air voids, enhancing mixture compactness and durability;
5. At 8% NM content, NS greatly enhances the Tensile Strength Ratio (TSR), achieving a value of 91.0%, well above the 80% minimum standard. NA and NT also show improved moisture resistance, reaching TSRs of 88.0% and 84.1%, respectively. These results highlight the efficiency of nanomaterials in enhancing the asphalt mixes’ resistance to moisture-induced damage;
6. The addition of nanomaterials at 8% NM content notably reduces permanent deformation, with NA, NS, and NT decreasing permanent strain by approximately 69%, 82%, and 64% at 10,000 cycles, respectively, compared to the control mix. These results demonstrate the effective role of nanomaterials in reducing strain accumulation, substantially increasing the longevity and structural stability of asphalt pavements;
7. For fatigue resistance, NS, NA, and NT enhance the asphalt’s ability to resist fatigue cracking, particularly at nanomaterial contents of 4% for NS and 6% for NA and NT. The IDEAL-CT test results show that at these contents, NS, NA, and NT improve fatigue life by 14.6%, 17.5%, and 20.5%, respectively, compared to the control mix;
8. Based on the comprehensive experimental analysis reported in this investigation, it is recommended to adopt a 4% NM content for NS and a 6% NM content for both NA and NT. This recommendation is due to their superior performance against various types of pavement distresses at these concentrations. The experimental results summarized in Table 8 compare the essential characteristics of the asphalt binder and mixes at the recommended dosages and the reference binder as well as the reference mix (0% NMs). While the experimental results are promising, the successful application of these nanomaterial concentrations in practical asphalt paving applications has to be confirmed by additional field testing. This step is crucial for ensuring that the laboratory findings translate effectively to actual performance improvements in the community of asphalt paving work.

## Figures and Tables

**Table 1:** Characteristics of the asphalt cement used in this investigation.

Type of Test	Unit	Specifications	Results	Limits [31]
Penetration at 25 °C, 100 g, and 5 s	0.1 mm	ASTM D5 [33]	49	40–50
Softening point, ring, and ball	°C	ASTM D36 [34]	49.7	----
Specific gravity at 25 °C	----	ASTM D70 [35]	1.034	----
Flashpoint	°C	ASTM D92 [36]	294	>232
Ductility	cm	ASTM D113 [37]	120	>100
Residue from thin film oven test				
Retained penetration from original	%	ASTM D5 [33]	61	>55
Ductility at 25 °C, 5 cm/min	cm	ASTM D113 [37]	70	>25

**Table 2:** Physical characteristics of the aggregate utilized in this study.

Material and Property	Specifications	Results	Limits [31]
Coarse Aggregate			
Apparent Specific Gravity	C127 [38]	2.712	-
Bulk Specific Gravity		2.632	-
Water Absorption (%)		0.402	-
Soundness (Sodium Sulfate Solution Loss) (%)	C88 [39]	4.2	≤12
Percent Wear (Los Angeles abrasion) (%)	C131 [40]	19	≤30
Flat and Elongated (5:1) (%)	D4791 [41]	4	≤10
Fractured Pieces (%)	D5821 [42]	94	≤90
Fine Aggregate			
Apparent Specific Gravity	C128 [43]	2.583	-
Bulk Specific Gravity		2.522	-
Water Absorption (%)		0.933	-
Clay Lump and Friable Particles (%)	C142 [44]	1.87	≤3
Sand Equivalent (%)	D2419 [45]	61	≥45

**Table 3:** Selected aggregate gradation and specification limit.

Size of Sieve (mm)	19	12.5	4.75	2.36	0.3	0.075
Gradation, %	100	95	59	37	13	7
ASTM D3515, D-5 limit, %	100	90–100	44–74	28–58	5–21	4–10

**Table 4:** Chemical composition and physical properties of mineral filler.

Chemical Composition, %						
CaO	SiO_2_	Al_2_O_3_	MgO	Fe_2_O_3_	SO_3_	L.O.I
65.3	8.12	1.32	0.16	0.33	0.54	24.23
Physical Properties						
Specific Gravity		Surface Area * (m^2^/kg)		Passing Sieve No. 200 (0.075), %		
2.76		241		97		

* Blain air permeability method.

**Table 5:** Physical properties of nanomaterials.

Properties	Nanomaterial		
	NA	NS	NT
Chemical formula	Al_2_O_3_	SiO_2_	TiO_2_
Molecule Wt. (g/mol)	101.96	60.08	85.42
Appearance	White powder	Pearl-white powder	Off-white powder
Average particle size (nm)	10–20	25–35	20–30
Purity (%)	99.9	99.8	99.9
Specific surface area (m^2^/gm)	120–160	190–250	120–160
Melting point (°C)	2030	1730	1860
Bulk density (g/mL)	0.2	0.08	0.51

**Table 6:** Effect of NMs on the viscosity of asphalt binder.

		Original			After RTFO		
Temperature, °C		115	135	155	115	135	155
NM Type	Content, %	Viscosity, mPa·s					
RB	0	2268	719.7	217.8	2983	996.7	319.2
NA	2	2753.3	845.2	291.2	3532	1080	388.3
	4	2917	965.9	331.4	3400	1200	404.8
	6	3153	1063.0	350.4	4340	1307	480.6
	8	3596	1089	388.3	4460	1494	537.4
NS	2	2651.5	804.9	262.8	3768.9	1124.5	393
	4	3584.3	1129.3	419	4469.7	1285.5	435.6
	6	3851.8	1325.8	447.4	5456.9	1751.9	655.8
	8	5416.7	1770.8	639.2	7036	2331.9	897.3
NT	2	2578.1	793.1	255.7	3127.4	1044	383.5
	4	2836.2	904.4	298.3	3367.2	1197.9	393
	6	2971.1	916.8	317.2	4280	1231.1	447.4
	8	2997.2	982.5	336.2	4417	1319.2	459.3

**Table 7:** Impact of NMs on permanent deformation parameters.

NM Content, %	NM Type	Permanent Deformation Parameters		
		a	b	(*ε_p_*) at N = 10,000
0	-	201	0.3974	7813
2	NA	211	0.3786	6897
	NS	198	0.3745	6233
	NT	182	0.3871	6521
4	NA	188	0.356	5120
	NS	152	0.3221	3120
	NT	201	0.3742	6100
6	NA	160	0.331	3600
	NS	132	0.2974	2130
	NT	185	0.347	4400
8	NA	142	0.312	2410
	NS	112	0.2644	1380
	NT	165	0.302	2800

**Table 8:** Key properties of unmodified and modified binder and mixes with NMs.

**Properties**	**Asphalt Binder Level**			
	**RB**	**4% NS**	**6% NA**	**6% NT**
Penetration (1/10 mm)	49	43	41	42
Viscosity @ 135 °C (mPa·s)	719.7	1129.3	1063.0	916.8
Softening point (°C)	49.7	52.1	52.9	53.1
Mass loss (%)	0.266	0.321	0.303	0.238
∆T (°C)	0.4	0.7	1.3	1.7
**Properties**	**Asphalt Concrete Level**			
	**CM**	**4% NS**	**6% NA**	**6% NT**
Marshall stability (KN)	9.84	11.32	11.24	10.72
Flow value (mm)	3.35	2.85	2.74	2.98
AV (%)	4	3.35	3.65	3.98
VMA (%)	14.52	14.21	15.12	15.64
VFA (%)	72.56	76.43	75.86	74.55
TSR (%)	77	83	85.1	82.2
Mr (MPa)	2707	2916	2932	2846
(*ɛ_p_*) at N = 10,000 microstrain	7813	3120	3600	4400
CT index	55.12	63.21	64.81	66.47

## Data Availability

The original contributions presented in the study are included in the article, further inquiries can be directed to the corresponding author.
